# Integrative Clinical and Functional Characterization of the *TCF7L2* rs7903146 Variant Reveals Regulatory Mechanisms Linking Genetic Susceptibility to Oxidative Stress in Type 2 Diabetes

**DOI:** 10.3390/diagnostics16152423

**Published:** 2026-07-31

**Authors:** Ahmed M. Ahmed, Hakeemah H. Al-Nakhle, Amjad M. Yousuf, Hamza M. A. Eid, Abdel Rahim M. Muddathir, Awadh S. Alsubhi, Hashim M. Aljohani, Renad M. Alhamawi, Mustafa Y. Taher, Faisal Almalki, Kholoud Ashour, Yahya A. Almutawif

**Affiliations:** 1 Department of Clinical Laboratory Sciences, College of Applied Medical Sciences, Taibah University, Madinah 42353, Saudi Arabia; hnakhly@taibahu.edu.sa (H.H.A.-N.); amyousef@taibahu.edu.sa (A.M.Y.); heid@taibahu.edu.sa (H.M.A.E.); ammahmoud@taibahu.edu.sa (A.R.M.M.); assubhi@taibahu.edu.sa (A.S.A.); hsnani@taibahu.edu.sa (H.M.A.); rhamawi@taibahu.edu.sa (R.M.A.); mtaher@taibahu.edu.sa (M.Y.T.); fmalki@taibahu.edu.sa (F.A.); kaashour@taibahu.edu.sa (K.A.); ymutawif@taibahu.edu.sa (Y.A.A.); 2 Health and Life Research Center, Taibah University, Madinah 42353, Saudi Arabia

**Keywords:** *TCF7L2*, rs7903146, type 2 diabetes mellitus, T2DM, oxidative stress, eQTL, regulatory variant

## Abstract

**Background:** The transcription factor 7-like 2 (*TCF7L2*) gene is one of the strongest genetic determinants of type 2 diabetes mellitus (T2DM), with the rs7903146 (C > T) polymorphism consistently associated with impaired insulin secretion and glucose dysregulation. This study investigated the associations between the *TCF7L2* rs7903146 polymorphism, glycemic control, oxidative stress biomarkers, and T2DM susceptibility, integrating bioinformatic analyses to explore the functional significance of this variant. **Methods:** This case–control study included 200 patients with T2DM, 100 prediabetic individuals, and 120 healthy controls. Genotyping of rs7903146 was performed using TaqMan SNP assays. Biochemical analyses included fasting plasma glucose (FPG), glycated hemoglobin (HbA1c), lipid profile, and oxidative stress biomarkers, including superoxide dismutase (SOD), glutathione peroxidase (GPx), total antioxidant capacity (TAC), and malondialdehyde (MDA). Bioinformatic analyses included population frequency analysis, regulatory annotation, chromatin accessibility assessment, expression quantitative trait locus (eQTL) analysis, protein interaction network construction, and pathway enrichment analyses to investigate the functional consequences of rs7903146. **Results:** The T allele frequency was markedly higher in T2DM patients (33.5%) and prediabetic individuals (30%) than in controls (13.3%) (*p* < 0.001). T2DM susceptibility increased under allelic (OR = 3.27, 95% CI: 2.14–5.01), dominant (OR = 3.9, 95% CI: 2.37–6.42), and recessive (OR = 6.92, 95% CI: 1.59–30.07) models (*p* < 0.01). T2DM patients also showed significantly lower superoxide dismutase (SOD) and glutathione peroxidase (GPx) activities, lower total antioxidant capacity (TAC), and higher MDA levels (*p* < 0.001). T allele carriers had poorer glycemic control and greater oxidative stress. Bioinformatic analyses showed that rs7903146 resides within an active intronic regulatory region with chromatin accessibility, enhancer-associated histone marks, transcription factor occupancy, and candidate cis-regulatory elements. eQTL analyses showed tissue-specific effects on *TCF7L2* expression, while network and pathway analyses highlighted WNT signaling, β-catenin transcriptional complexes, and metabolic regulation and oxidative stress pathways. **Conclusions:** The TCF7L2 rs7903146 polymorphism was significantly associated with T2DM susceptibility, impaired glycemic regulation, and altered oxidative stress biomarkers. Bioinformatic analyses provided predictive evidence suggesting that rs7903146 may have tissue-specific regulatory relevance and may be indirectly linked to metabolic and WNT/β-catenin signaling pathways. However, because of the observational case–control design, these findings do not establish causality, and the proposed regulatory mechanisms require experimental validation.

## 1. Introduction

Type 2 diabetes mellitus (T2DM) is a complex metabolic disease characterized by persistent hyperglycemia resulting from decreased insulin production and insulin resistance. Due to the interplay between genetic vulnerability and environmental factors, including sedentary lifestyles and dietary choices, the worldwide incidence of T2DM continues to rise rapidly [1]. A major contributor to morbidity and mortality globally, poor glycemic management is closely linked to the emergence of microvascular and macrovascular problems.

The pathophysiology of T2DM is significantly influenced by genetic background. According to genome-wide association studies, the transcription factor 7-like 2 (*TCF7L2*) gene is one of the most important susceptibility genes for type 2 diabetes [2]. *TCF7L2* gene is an essential transcription factor in the classical WNT/β-catenin signaling pathway, which regulates insulin production, glucose homeostasis, and pancreatic β-cell activity. One of its genetic variants, the intronic rs7903146 (C > T) polymorphism, has been frequently associated with an increased risk of type 2 diabetes mellitus (T2DM) across numerous populations. Changes in this gene have been associated with altered glucose metabolism and decreased insulin production.

One of the most extensively studied genetic variants linked to T2DM is the single-nucleotide polymorphism (SNP) rs7903146 (C/T) in the *TCF7L2* gene. This polymorphism has been strongly linked to an elevated risk of type 2 diabetes in a range of groups, according to numerous studies and systematic reviews [1]. T allele carriers are more likely to have poor insulin production, decreased β-cell activity, and heightened vulnerability to metabolic dysregulation. Furthermore, diabetic nephropathy and other problems connected to diabetes have been linked to this variation [3].

Oxidative stress is an important contributor to the development of T2DM and its consequences, alongside genetic factors. Excessive formation of reactive oxygen species (ROS) is encouraged by chronic hyperglycemia, which worsens insulin resistance and causes cellular damage and β-cell malfunction. The pathophysiology of diabetes and its related problems is influenced by this imbalance between pro-oxidants and antioxidants. To evaluate the degree of oxidative stress in patients with diabetes, biomarkers such as superoxide dismutase (SOD), glutathione peroxidase (GPx), total antioxidant capacity (TAC), and malondialdehyde (MDA) are often used [4].

Few studies have examined the associations between *TCF7L2* polymorphisms and oxidative stress biomarkers and glycemic control, despite a wealth of literature on their associations with T2DM risk. According to recent research, the rs7903146 mutation may affect oxidative processes, lipid metabolism, and glucose regulation [5]. Even so, it remains unknown how precisely this genetic variant is linked to oxidative stress. Thus, the goal of the current study was to explore the relationship between the *TCF7L2* rs7903146 polymorphism and the risk of T2DM, as well as its associations with oxidative stress biomarkers and glycemic control, in patients with T2DM and prediabetes compared with healthy controls.

## 2. Materials and Methods

### 2.1. Study Design and Participant

200 patients with T2DM, 100 people with prediabetes, and 120 healthy controls participated in this case–control study during the period from 1 April 2025 to 31 December 2025. Participants were enrolled from many hospitals in Madinah, Kingdom of Saudi Arabia. The American Diabetes Association (ADA) criteria for diagnosing T2DM were HbA1c ≥ 6.5% and fasting plasma glucose (FPG) ≥ 7.0 mmol/L. In contrast, for prediabetes, the criteria were HbA1c values of 5.7–6.4% and FPG 6.6–6.9 mmol/L. In addition, participants of Saudi ethnicity who were at least eighteen years old were eligible. ADA criteria were used to diagnose T2DM and prediabetes categories. Age- and sex-matched people without a history of diabetes who had normal fasting plasma glucose and HbA1c levels served as healthy controls. The study excluded those with type 1 diabetes, pregnancy, autoimmune or chronic inflammatory illnesses, cancer, severe hepatic or renal impairment, or acute infections. Moreover, control subjects were enrolled based on the absence of diabetes or any history of metabolic disease. The ADA inclusion criteria required normal fasting plasma glucose (FPG) and HbA1c levels, as well as no prior diagnosis of type 2 diabetes. Individuals with HbA1c ≥ 6.5% or FPG ≥ 7.0 mmol/L were not included. Additionally, individuals having a history of diabetes, heart illness, chronic renal disease, inflammatory conditions, or the use of drugs that lower blood sugar were not allowed to participate. To verify that they were not diabetics, all controls underwent screening for diabetes-related metrics, such as fasting plasma glucose and HbA1c [6]. Medication use was not included as a covariate in the statistical analysis because information on the type, dosage, and duration of antidiabetic drugs was not systematically collected as part of the study protocol.

Exclusion criteria include patients with severe illnesses, cancer, liver problems, known autoimmune disorders, and end-stage kidney diseases that require dialysis. In addition, pregnant women, people with diabetes other than T2DM, and children were also excluded. The diagnosis of diabetes complications was established as previously mentioned [7]. A glomerular filtration rate (GFR) < 60 mL/min and a urine albumin-to-creatinine ratio (UACR) > 30 mg/g were used to diagnose nephropathy [8]. Furthermore, fundus findings (haemorrhages, microaneurysms, and neovascularization) from retinal imaging were used to diagnose retinopathy [9]. According to the American Academy of Neurology, neuropathy also includes clinical symptoms and nerve conduction testing [10]. Additionally, clinical and laboratory markers were used in accordance with ADA guidelines to diagnose cardiovascular diseases (CVD) [11].

### 2.2. Sample Size

Quanto software (version 1.2.4; University of Southern California, Los Angeles, CA, USA) was used to estimate the required sample size for an unmatched case–control genetic association study. The minimum required sample size was approximately 150 individuals, based on a two-sided α level of 0.05, 80% statistical power, an expected minor allele frequency of approximately 0.13 in the control group, and an expected odds ratio of approximately 3.0 for the rs7903146 variant. With 320 individuals in the primary case–control comparison (200 T2DM patients and 120 healthy controls), our study had statistical power greater than 90%. Furthermore, 100 people with prediabetes were included in secondary comparative studies.

### 2.3. Ethical Approval

The “Research Ethics Committee” at Taibah University granted permission for this study (Approval No. CLS 2020129). The “1964 Helsinki Declaration and its recent amendments” guided the conduct of this study. Before taking part in the study, each subject signed an informed consent form.

### 2.4. Biochemical Analysis

A Dimension^®^ EXLTM 200 comprehensive biochemistry autoanalyzer (Siemens, Erlangen, Germany) was used to enzymatically assess glucose and lipid profiles. In addition, A Bio-Rad D-10TM Hemoglobin Analyzer (Bio-Rad Laboratories, Hercules, CA, USA) was used to measure HbA1c.

### 2.5. Determination of Oxidative Stress Biomarkers

Following the manufacturer’s instructions, commercially available assay kits were used to measure oxidative stress indicators in serum samples. A colorimetric assay kit from (Cayman Chemical, Ann Arbor, MI, USA) (Product No. 706002) was used to evaluate superoxide dismutase (SOD) activity. An enzymatic test kit from (Randox Laboratories Ltd., Crumlin, UK) (Product No. RS504) was used to measure glutathione peroxidase (GPx) activity. A colorimetric test kit from (BioVision Inc., Milpitas, CA, USA) (Product No. K274) was used to measure total antioxidant capacity (TAC). A commercial kit from (Sigma-Aldrich, St. Louis, MO, USA) (Product No. MAK085) was used to measure malondialdehyde (MDA) levels as thiobarbituric acid reactive substances (TBARS). Using a UV-visible spectrophotometer (UV-1900i, Shimadzu Corporation, Kyoto, Japan), all measurements were carried out in accordance with the manufacturer’s instructions. The data were reported as U/mL for SOD, U/L for GPx, mmol/L for TAC, and nmol/mL for MD.

### 2.6. DNA Extraction and Genotyping Analysis

Peripheral venous blood samples (3–5 mL) were taken from each participant in sterile EDTA tubes. The Pure Link TM Genomic DNA Mini Kit (Thermo Fisher Scientific, Waltham, MA, USA) (Product No. K182001) was used to extract genomic DNA according to the manufacturer’s instructions. In short, cell lysis, protein digestion, and DNA binding to a silica membrane column were performed after leukocytes were separated from whole blood. After being cleaned of impurities, DNA was eluted in nuclease-free water. A Nano Drop ND-1000 spectrophotometer (Thermo Fisher Scientific, Waltham, MA, USA) was used to measure DNA concentration and purity; samples with an A260/A280 ratio between 1.8 and 2.0 were deemed acceptable. Before further examination, all DNA samples were stored at −20 °C.

Using a TaqMan SNP Genotyping Assay (Applied Biosystems, Thermo Fisher Scientific, Waltham, MA, USA) (assay ID: C_29347861_10), as recently reported [12], the target context sequence was: TAGAGAGCTAAGCACTTTTTAGATA[C/T]TATATAATTTAATTGCCGTATGAGG. Using a probe-based SNP genotyping assay (TaqMan system) intended to identify allelic variation with fluorescent VIC- and FAM-labeled probes, the *TCF7L2* rs7903146 (C/T) polymorphism was genotyped. The QuantStudio TM 5 Real-Time PCR System (Applied Biosystems, Thermo Fisher Scientific, Waltham, MA, USA) (Product No. A28139) was used for real-time PCR amplification. TaqMan Genotyping Master Mix (2×), TaqMan SNP Genotyping Assay (20×), and 10–20 ng of genomic DNA was added to a total volume of 10 µL for each reaction, which was adjusted with nuclease-free water.

Thermal cycling conditions were 10 min of initial denaturation at 95 °C, 40 cycles of denaturation at 95 °C for 15 s, and 60 s of annealing/extension at 60 °C. QuantStudio Design and Analysis Software was used to automatically perform allelic discrimination (CC, CT, and TT genotypes) based on endpoint fluorescence clustering. To reduce bias, all laboratory staff were blinded to case/control status, and as a quality-control measure, 10% of samples were randomly selected for repeat genotyping, yielding 100% concordance.

### 2.7. Statistical Analysis

SPSS software (version 23, IBM Corp., Armonk, NY, USA) was used for statistical analysis. While categorical data were presented as frequencies and percentages, continuous variables were reported as mean ± standard deviation (SD). *t*-test/One-way ANOVA was used to evaluate group differences, and post hoc tests were used for multiple comparisons. The chi-square (χ^2^) test and Fisher’s exact test were used to compare genotype and allele frequencies. By computing odds ratios (ORs) and 95% confidence intervals (CIs) under various genetic models (allelic, dominant, and recessive), the relationship between genotypes and T2DM risk was assessed. In addition, an adjusted model for age, BMI, sex, smoking status, hypertension, hyperlipidemia, and the dominant genetic model (TT + CT) was assessed. Statistical significance was defined as a *p* < 0.05.

### 2.8. Bioinformatic Analysis

#### Functional Annotation and Regulatory Characterization of rs7903146

To investigate the potential functional consequences of the TCF7L2 rs7903146 polymorphism and support clinical findings, a multi-layer bioinformatic analysis was performed. Population allele frequency data were obtained from publicly available reference databases, including ALFA, gnomAD v4, 1000 Genomes Project (1000G 30×), and PAGE, to investigate ancestry-specific distribution patterns.

Regulatory annotation of rs7903146 was conducted using RegulomeDB (https://regulomedb.org/regulome-search, accessed on 30 May 2026) to evaluate chromatin accessibility, transcription factor binding, DNase hypersensitivity, and candidate cis-regulatory element (cCRE) overlap. The genomic position of rs7903146 within the *TCF7L2* locus was visualized using GRCh38 genomic coordinates with GENCODE transcript annotations.

Chromatin accessibility was assessed using DNase I hypersensitivity and ATAC-seq datasets, particularly in pancreatic tissue, whereas enhancer-associated histone modifications (H3K4me1) and transcription factor occupancy data were extracted from ENCODE and related epigenomic resources.

Expression quantitative trait locus (eQTL) analysis was performed using GTEx datasets to evaluate the effect of rs7903146 on *TCF7L2* expression across metabolically relevant tissues, including subcutaneous adipose tissue, visceral adipose tissue (omentum), arterial tissues, heart tissues, liver, pancreas, and whole blood (https://gtexportal.org/home/eqtlDashboardPage, accessed on 30 May 2026). Statistical significance was determined using GTEx eQTL association statistics.

Protein interaction networks were generated using STRING to identify functional relationships between *TCF7L2* and oxidative stress-associated genes, including *SOD1, SOD2*, *GPX1*, *GPX3*, *CAT*, *NFE2L2*, *TXNIP*, *NOX4*, and *PRDX* family members. Functional enrichment analyses were subsequently performed using KEGG and Reactome databases to identify enriched biological pathways (https://string-db.org/, accessed on 30 May 2026).

## 3. Results

### 3.1. Baseline Characteristics of Study Population

This study included 420 participants in total: 200 patients with type 2 diabetes mellitus (T2DM), 100 people with prediabetes, and 120 healthy controls. There were 129 males (64.5%) and 71 females (35.5%) in the T2DM group, 69 males (69.0%) and 31 females (31.0%) in the prediabetes group, and 71 males (59.2%) and 49 females (40.8%) in the control group. The T2DM group’s mean age was 51.2 ± 12.1 years, the prediabetes group was 41.9 ± 13.2 years, and the control group was 45.1 ± 10.5 years. Participants’ clinical features and demographics are listed in Table 1. There was no significant difference in gender (*p* > 0.05). In addition, compared to prediabetic participants and controls, T2DM patients were older than both the prediabetes and control groups (*p* < 0.001). The three groups’ BMIs varied significantly (*p* < 0.001), with the prediabetes group having the highest mean BMI, followed by the T2DM group, and the healthy controls having the lowest. Moreover, T2DM and prediabetes were found to have elevated glycemic markers (FPG, HbA1c) (*p* < 0.001). Furthermore, patients with T2DM had higher levels of TC, TG, and LDL, and lower levels of HDL (*p* < 0.05), and showed considerably higher MDA levels and significantly lower activities of the antioxidant enzymes SOD, GPx, and TAC compared with the prediabetes and control groups (*p* < 0.001).

### 3.2. Distribution of TCF7L2 rs7903146 Genotypes and Alleles

Table 2 presents the distribution of TCF7L2 rs7903146 genotypes and alleles. The study groups differed significantly (*p* < 0.001). In addition, the CC genotype was increasingly prevalent in the control group, whereas the TT and CT genotypes were more common in the T2DM and prediabetes groups. Moreover, the frequency of the T allele was significantly higher in T2DM and prediabetes than in controls (*p* < 0.001).

### 3.3. Hardy–Weinberg Equilibrium Study of TCF7L2 rs7903146 Genotypes

Table 3 shows that genotype distributions were in line with the Hardy–Weinberg equilibrium, with no discernible deviation (*p* > 0.05).

### 3.4. Pairwise Comparisons Across Study Population

Table 4 summarizes the significant differences in genotype and allele distributions between the T2DM and control, and prediabetes and control, groups, as identified by pairwise comparisons (*p* < 0.001). However, there were no statistically significant differences between the T2DM and prediabetes groups (*p* > 0.05).

### 3.5. Association of TCF7L2 (rs7903146) Genotypes with Clinical, Demographic, and Biochemical Profiles

Table 5 shows the association between TCF7L2 (rs7903146) and risk factors, including clinical, demographic, and biochemical profiles, across all study groups. Most demographic factors, such as gender, BMI, smoking status, and hypertension, did not significantly correlate with the relationship between TCF7L2 rs7903146 genotypes and clinical features (*p* > 0.05). Nonetheless, a strong association (*p* = 0.009) between the TT genotype and cardiovascular disease was observed in T2DM patients. Biochemically, carriers of the T allele (TT and CT genotypes) had significantly higher FPG and HbA1c levels than CC carriers in both the T2DM and prediabetes groups (*p* < 0.05). Moreover, T allele carriers in the T2DM group showed considerably lower SOD activity and total antioxidant capacity (TAC), and significantly higher MDA levels (*p* < 0.05).

### 3.6. Genetic Model Analysis of the Relationship Between T2DM Susceptibility and TCF7L2 rs7903146

Table 6 shows a strong correlation between TCF7L2 rs7903146 and T2DM risk across multiple genetic models. In the allelic model, the T allele was associated with a markedly higher risk of T2DM (OR = 3.27, 95% CI: 2.14–5.01, *p* < 0.001). Both the recessive model (OR = 6.92, 95% CI: 1.59–30.07, *p* = 0.002) and the dominant model (TT + CT vs. CC) also showed a robust association (OR = 3.90, 95% CI: 2.37–6.42, *p* < 0.001).

### 3.7. Multivariable Logistic Regression Analyses Adjusting for Age, BMI, Sex, Smoking Status, Hypertension, and Hyperlipidemia Confounders

After controlling for age, BMI, sex, smoking status, hypertension, and hyperlipidemia, multivariable logistic regression analysis was performed (Table 7). T2DM remained independently associated with the TCF7L2 rs7903146 dominant model (TT + CT) (adjusted OR = 3.75, 95% CI: 2.60–5.51, *p* < 0.001). After adjustment, age, BMI, sex, smoking status, and hypertension were not significant, whereas hyperlipidemia was independently associated with T2DM (adjusted OR = 2.91, 95% CI: 1.81–4.89, *p* < 0.001).

### 3.8. Association Between TCF7L2 rs7903146 Genotype and Oxidative Stress Biomarkers

Table 8 presents the association between the TCF7L2 rs7903146 dominant genotype and decreased SOD and TAC levels, as well as increased MDA levels, remained statistically significant after controlling for glycemic status (HbA1c), age, BMI, sex, smoking status, and hypertension. However, the association with GPx was weakened and no longer significant.

### 3.9. Regulatory and Functional Characterization of rs7903146 Supports Its Role as a Functional Non-Coding Variant

**Figure 1:**
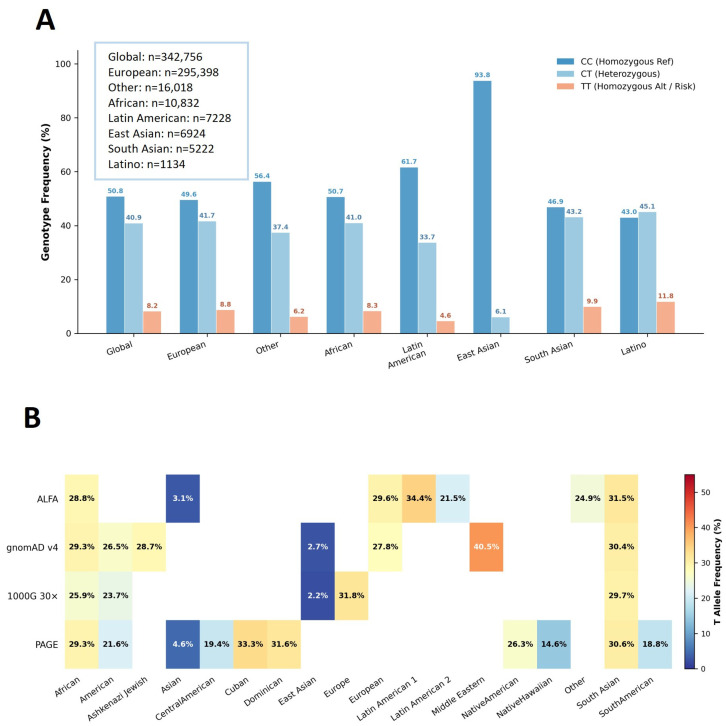
Population distribution and ancestry-specific variation in rs7903146 (C > T) frequencies across reference populations. (**A**) Distribution of rs7903146 genotypes (CC, CT, and TT) across major ancestry groups, with frequencies estimated under Hardy–Weinberg equilibrium using publicly available population datasets. (**B**) A heatmap comparing T allele frequencies across ancestry groups from ALFA, gnomAD v4, 1000 Genomes Project (1000G 30×), and PAGE databases. Color intensity reflects T allele frequency, ranging from low (blue) to high (red). The results demonstrate substantial ancestry-dependent variation in rs7903146, emphasizing the importance of population stratification in genetic association studies involving TCF7L2.

Population frequency analysis revealed considerable ancestry-dependent variation in the distribution of rs7903146, with substantially lower T allele frequencies in East Asian populations and higher frequencies in European, African, and South Asian populations, highlighting population-specific susceptibility patterns (Figure 1).

### 3.10. Regulatory Annotation and Chromatin Accessibility Analysis of TCF7L2 rs7903146

**Figure 2:**
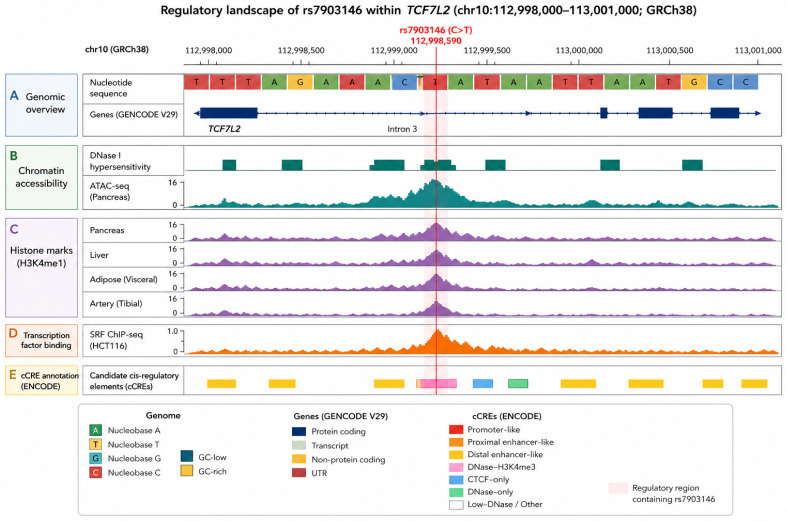
Multi-layer regulatory annotation of rs7903146 within the *TCF7L2* locus. (**A**) shows the genomic position of rs7903146 (C > T) within intron 3 of *TCF7L2* (GRCh38). (**B**) illustrates chromatin accessibility using DNase hypersensitivity and pancreatic ATAC-seq data, demonstrating localization within accessible chromatin. (**C**) presents enhancer-associated histone marks (H3K4me1) across relevant tissues, showing overlap with the variant position. (**D**) displays SRF ChIP-seq evidence of transcription factor occupancy, while (**E**) shows ENCODE candidate cis-regulatory element (cCRE) annotations overlapping rs7903146.

Regulatory annotation revealed that rs7903146 is located within intron 3 of TCF7L2 and overlaps multiple regulatory features indicative of functional activity. Chromatin accessibility analysis demonstrated that the variant resides in regions with DNase hypersensitivity and pancreatic ATAC-seq peaks, indicating it is within accessible chromatin. Furthermore, enhancer-associated histone modifications (H3K4me1) overlapped the variant position across multiple metabolically relevant tissues, including pancreas, liver, adipose tissue, and tibial artery, supporting enhancer-like regulatory activity (Figure 2).

### 3.11. Tissue-Specific eQTL Effects of TCF7L2 rs7903146 on Gene Expression

**Figure 3:**
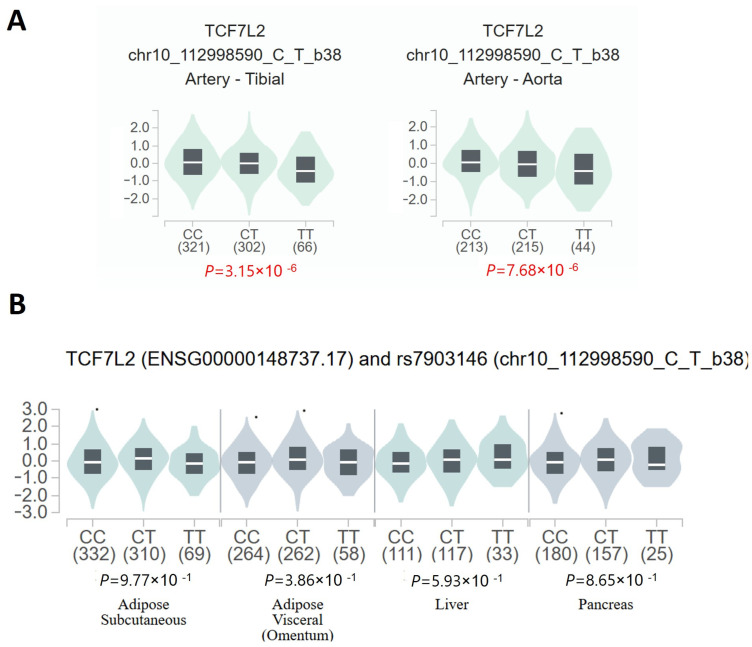
Tissue-specific association of the TCF7L2 rs7903146 variant with TCF7L2 expression. Violin plots show normalized TCF7L2 expression according to rs7903146 genotype (CC, CT, and TT) across selected GTEx tissues. (**A**) Significant genotype-dependent differences were observed in tibial artery (*p* = 3.15 × 10^−6^) and aorta (*p* = 7.68 × 10^−6^), with the TT genotype exhibiting lower TCF7L2 expression. (**B**) No significant genotype-associated differences were detected in subcutaneous adipose tissue (*p* = 0.977), visceral adipose tissue–omentum (*p* = 0.386), liver (*p* = 0.593), or pancreas (*p* = 0.865). Pale green and light blue-gray violins represent the expression distributions, whereas the dark gray internal box plots indicate the median and interquartile range. Violin width reflects data density, and sample sizes for each genotype group are shown in parentheses. Significant *p* values are highlighted in red. The rs7903146 variant is represented in GTEx as chr10_112998590_C_T_b38.

eQTL analysis demonstrated tissue-specific effects of rs7903146 on TCF7L2 expression. Significant associations were observed in vascular tissues, with TT genotype carriers showing reduced TCF7L2 expression in both the tibial artery and aortic tissues. In contrast, no significant differences in expression were observed in the pancreas, liver, visceral adipose tissue, or subcutaneous adipose tissue, suggesting context-dependent regulatory mechanisms (Figure 3).

### 3.12. Analysis of TCF7L2’s Protein–Protein Interaction Network and Related Regulatory Pathways

**Figure 4:**
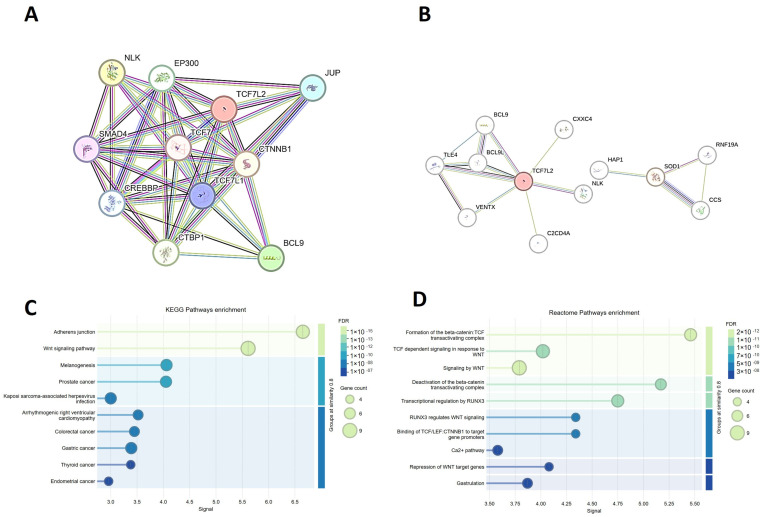
*TCF7L2* interaction networks and pathway enrichment. (**A**,**B**) STRING protein–protein interaction networks showing direct and indirect *TCF7L2*-associated proteins. Nodes represent proteins, and colored edges indicate different sources of interaction evidence. (**C**,**D**) KEGG and Reactome enrichment analyses of the associated proteins, respectively. Bubble size indicates gene count, bubble color represents the false-discovery rate (FDR), and the x-axis shows enrichment signal.

Protein interaction network analysis identified TCF7L2 as a central regulatory hub, primarily connected to canonical WNT signaling components, including CTNNB1, TCF7, TCF7L1, EP300, BCL9, CREBBP, and SMAD4. Although direct physical interactions between TCF7L2 and antioxidant enzymes were not identified, network analysis revealed indirect functional relationships linking TCF7L2 to oxidative stress pathways through intermediaries (Figure 4).

Functional enrichment analysis revealed significant enrichment of WNT signaling pathways, β-catenin transcriptional complexes, adherens junction pathways, RUNX signaling, and transcriptional regulation pathways. Collectively, these findings support the hypothesis that rs7903146 is a functional regulatory variant that alters transcriptional regulation and metabolic signaling, thereby potentially contributing to the dysregulation of oxidative stress observed in T-allele carriers.

## 4. Discussion

The current study examined the association among oxidative stress biomarkers, glycemic control, and the TCF7L2 rs7903146 polymorphism in patients with T2DM and prediabetes. Our findings demonstrate significant alterations in glycemic and oxidative stress markers, as well as a strong association between the T allele and an increased risk of type 2 diabetes.

Compared with controls, T2DM patients had higher baseline values for age, BMI, fasting glucose, and HbA1c. This aligns with the known contributions of aging and adiposity to the development of T2DM and insulin resistance [13,14]. The dyslipidemia observed in T2DM patients, characterized by elevated TC, TG, and LDL and reduced HDL, further supports the metabolic abnormalities linked to insulin resistance and prolonged hyperglycemia [15].

One key outcome of the study is a significant increase in oxidative stress among T2DM patients, as evidenced by reduced activities of the antioxidant enzymes SOD and GPx, lower TAC, and higher MDA levels. These results align with recent studies showing that prolonged hyperglycemia increases the production of reactive oxygen species (ROS), leading to oxidative damage and β-cell dysfunction [16,17]. Intermediate levels observed in prediabetic individuals suggest that oxidative stress begins early in the course of the disease, even before overt diabetes manifests, according to recent longitudinal investigations [18].

The T allele was significantly more prevalent in the T2DM and prediabetes groups than in the control group, as indicated by the *TCF7L2* rs7903146 genotype distribution. This outcome is in line with recent meta-analyses and genome-wide association studies that have identified rs7903146 as one of the most important genetic variations linked to the risk of type 2 diabetes in a variety of groups [1,19]. The lack of deviation from the Hardy–Weinberg equilibrium provides additional evidence for the reliability of the genotyping data.

Pairwise analysis showed significant differences in genotype and allele frequencies between each disease group and healthy controls, but no significant differences were discovered between the T2DM and prediabetes groups. This result suggests that the TCF7L2 rs7903146 mutation may exert a genetic effect early in the development of dysglycemia, before prediabetes progresses to established T2DM. This supports the theory that TCF7L2 variants contribute to early glucose dysregulation, as the genetic predisposition conferred by the T allele may already be established at the prediabetic stage [20].

Significantly higher FPG and HbA1c levels were observed in carriers of the T allele (TT and CT genotypes), indicating poorer glycemic control. This outcome is in line with recent research showing rs7903146 affects β-cell activity and insulin production instead of insulin sensitivity [19]. Mechanistically, TCF7L2 influences insulin secretion and incretin signaling by regulating proglucagon production and participating in the WNT signaling cascade.

Another significant finding is the association between oxidative stress indicators and the T allele. T allele carriers showed decreased SOD activity and TAC, and increased lipid peroxidation (MDA), indicating that this genetic variation may worsen oxidative stress. According to recent studies, genetic variations related to metabolic control, such as TCF7L2, may interact with antioxidant defense systems and oxidative stress pathways, contributing to insulin resistance and diabetes risk, although few studies have specifically examined this relationship [21]. This suggests a potential mechanistic connection between oxidative stress and genetic vulnerability in type 2 diabetes.

Additionally, in T2DM patients, a strong correlation was found between the TT genotype and cardiovascular illnesses. This conclusion is corroborated by new research indicating that variations in the *TCF7L2* gene may increase the risk of both diabetes and cardiovascular problems through pathways including oxidative stress, inflammation, and endothelial dysfunction [22,23,24]. This demonstrates *TCF7L2’s* wider involvement in vascular and metabolic dysfunction.

Genetic model analysis showed that the T allele and T2DM risk were strongly correlated under allelic, dominant, and recessive models, with the recessive models showing the highest risk. These results are consistent with other extensive meta-analyses that have supported the strong impact of rs7903146 on T2DM susceptibility [1,25].

Our clinical findings demonstrated that carriers of the rs7903146 T allele exhibited significantly poorer glycemic control, characterized by higher fasting glucose and HbA1c levels, together with reduced antioxidant defenses and increased lipid peroxidation. These findings are consistent with extensive genetic evidence indicating that rs7903146 is among the strongest common susceptibility loci associated with impaired glucose homeostasis, reduced insulin secretion, and increased risk of type 2 diabetes across diverse populations [19,26,27,28,29]. Although our cross-sectional case–control design does not permit causal inference, these observations indicate that rs7903146 T-allele carriage is associated with poorer glycemic regulation and an altered oxidative stress profile. The present data cannot determine whether the variant directly contributes to oxidative stress, whether the association is mediated through glycemic dysregulation, or whether residual clinical and environmental factors are involved [17,18,30].

By showing that rs7903146 is situated within an active intronic regulatory region marked by chromatin accessibility, enhancer-associated histone modifications, transcription factor occupancy, and potential cis-regulatory elements, bioinformatic analyses offer mechanistic support for these findings [31,32,33]. These results are in line with earlier functional research showing that rs7903146 functions mostly as a regulatory variable that affects enhancer activity and modifies TCF7L2 transcriptional control rather than as a protein-coding variant [31,32].

After controlling for HbA1c and other clinical variables, there are still strong correlations between the TCF7L2 rs7903146 dominant model and SOD, TAC, and MDA. This suggests that these correlations may be due to the genetic variant’s independent contribution to oxidative stress pathways rather than just variations in glycemic control. Crucially, when controlling for important clinical variables, the relationship between the *TCF7L2* rs7903146 mutation and T2DM remained significant, suggesting that the observed genetic association is unaffected by age, BMI, sex, smoking status, hypertension, and hyperlipidemia. These findings, however, show association rather than causation due to the observational approach, and the bioinformatic results should be regarded as supportive prediction evidence until they are validated experimentally.

Interestingly, tissue-specific regulatory effects were identified by eQTL analysis; high correlations were observed in vascular tissues but not in conventional metabolic locations such as the pancreas, liver, or adipose tissue. These findings suggest that the functional consequences of rs7903146 are likely context-dependent and mediated by tissue-specific regulatory mechanisms rather than resulting from broad alterations in gene expression. Such tissue specialization makes biological sense because chromatin accessibility and enhancer activity are strongly dependent on the cellular environment, particularly at metabolic regulation locations.

The observed decline in antioxidant markers among T-allele carriers may be supported by network and pathway analysis. Despite the absence of direct contacts between TCF7L2 and traditional antioxidant enzymes, protein interaction networks revealed indirect links between TCF7L2 and oxidative stress-related pathways through transcriptional regulation and canonical WNT signaling components [34,35]. Functional enrichment supported a model in which genetic variation in *TCF7L2* may indirectly contribute to oxidative imbalance through impaired metabolic homeostasis, mitochondrial dysfunction, altered glucose utilization, and subsequent reactive oxygen species generation rather than direct regulation of antioxidant enzymes. It also revealed significant involvement of metabolic signaling pathways and the β-catenin/TCF transcriptional complex [18,30,34,35,36].

After adjusting for multiple variables, hyperlipidemia continues to be an important predictor for T2DM. This upholds the link between dyslipidemia and insulin resistance. Nevertheless, after normalization, the association with GPx became statistically non-significant, indicating that GPx activity is more impacted by glycemic status and clinical variables than the rs7903146 genotype. It appears that the altered patterns of oxidative stress may be related to the *TCF7L2* rs7903146 variant and represent an additional independent genetic element, rather than the phenomenon being caused exclusively by hyperglycemia.

Collectively, the integration of the clinical and bioinformatic findings supports the hypothesis that rs7903146 may have regulatory relevance and may be indirectly linked to metabolic and oxidative stress-related pathways. These computational observations offer a potential biological framework for interpreting the associations among T-allele carriage, impaired glycemic regulation, and altered oxidative stress biomarkers. However, they do not demonstrate that rs7903146 directly causes these changes or confirm the proposed molecular pathways.

Several limitations should be acknowledged; the case–control design does not permit causal inference or establish temporal relationships. Residual confounding cannot be excluded, as the groups differed in BMI and metabolic profile and medication use was not available for adjustment. The single-center, single-ethnicity sample and the rarity of the (TT) genotype limit generalizability and power for gene–environment interactions, and the focus on a single variant does not capture broader polygenic effects. Finally, the proposed mechanisms rest on predictive bioinformatic analyses that were not experimentally validated; functional studies will be required for confirmation. Prospective, multi-center, and functional investigations are warranted.

## 5. Conclusions

Considering the limitations inherent to an observational case–control design, the findings of this study indicate significant associations, but do not establish causality, between the *TCF7L2* rs7903146 polymorphism, increased oxidative stress, and impaired glycemic control in individuals with type 2 diabetes mellitus. The oxidative imbalance was reflected by reduced SOD and GPx activities, decreased TAC, and elevated MDA levels. In addition, the bioinformatic analyses suggested potential tissue-specific regulatory effects involving metabolic pathways and WNT/β-catenin signaling. However, these computational findings remain predictive and require experimental validation. Further prospective and functional studies are therefore needed to confirm these associations, determine their causal relevance, and clarify the underlying molecular mechanisms.

## Figures and Tables

**Table 1:** Demographic, Clinical Characteristics, and Biochemical Parameters of Participants.

Parameters	Cases (*n* = 300)		Controls (*n* = 120)	*p*-Value
	T2DM (*n* = 200)	Prediabetes (*n* = 100)		
Gender (males, females)	129, 71	69, 31	71, 49	0.31
Age (years)	51.2 ± 12.1	41.9 ± 13.2	45.1 ± 10.5	<0.001 *
BMI	25.2 ± 3.3	26.3 ± 2.1	23.1 ± 1.3	<0.001 *
Blood pressure				
SBP (mm/Hg)	120.2 ± 15.5	119.5 ±11	119 ± 9.6	0.71
DBP (mm/Hg)	80.9 ± 5.1	80.5 ± 6.1	79.9 ± 2.9	0.21
Hypertensive patients	65 (32.5%)	22 (22%)	-	0.058
Smoking	71 (35.5%)	39 (39%)	31 (25.8%)	0.08
FPG (mmol/L)	7.2 ± 1.6	6.7 ± 0.9	5.01 ± 0.55	<0.001 *
HbA1c %	7.3 ± 2.29	5.9 ± 1.2	5.0 ± 0.6	<0.001 *
TC (mmol/L)	4.16 ± 0.29	3.91 ± 0.3	3.51 ± 0.1	<0.001 *
TG (mmol/L)	1.59 ± 0.8	1.47 ± 0.6	1.37 ± 0.3	0.012 *
LDL (mmol/L)	2.51 ± 0.9	2.36 ± 0.2	2.31 ± 0.2	0.016 *
HDL (mmol/L)	1.19 ± 0. 2	1.24 ± 0.1	1.25 ± 0.1	0.001 *
SOD (U/mL)	87.5 ± 15.1	112 ± 12.9	120.9 ± 11.8	<0.001 *
GPx (U/L)	41.9 ± 5.9	49.1 ± 5.9	59.1 ± 9.2	<0.001 *
TAC (mmol/L)	0.95 ± 0.26	1.15 ± 0.29	1.35 ± 0.2	<0.001 *
MDA (nmol/mL)	5.2 ± 1.1	4.6 ± 1.2	2.8 ± 0.9	<0.001 *
Nephropathy	17 (8.5%)	-	-	-
Retinopathy	11 (5.5%)	-	-	-
Cardiovascular diseases	15 (7.5%)	-	-	-
Neuropathy	8 (4%)	-	-	-

*: significant at *p* < 0.001. Abbreviations: *n*: number. BMI: Body mass index. SBP: systolic blood pressure. DBP: diastolic blood pressure. FPG: fasting plasma glucose. HbA1c: glycated hemoglobin. TC: total cholesterol. TG: triglyceride. LDL: low-density lipoprotein cholesterol. HDL: high-density lipoprotein cholesterol. SOD: Superoxide dismutase. GPx: Glutathione peroxidase. TAC: Total antioxidant capacity. MDA: Malondialdehyde.

**Table 2:** *TCF7L2* (rs7903146) genotype and allele distribution in the three study groups.

Genotype/Allele	T2DM Cases(*n* = 200) *n* (%)	Prediabetes Cases(*n* = 100) *n* (%)	Control(*n* = 120) *n* (%)	*p*-Value
Genotype (%)				
TT	21 (10.5%)	8 (8%)	2 (1.7%)	<0.001 *
CT	92 (46%)	44 (54%)	28 (23.3%)	
CC	87 (43.5%)	48 (38%)	90 (75%)	
TT + CT	113	52	30	
Allele (%)				
T	134 (33.5%)	60 (30%)	32 (13.3%)	<0.001 *
C	266 (66.5%)	140 (70%)	208 (86.7%)	

*: significant at *p* < 0.001. *n*: number.

**Table 3:** Hardy–Weinberg equilibrium for study groups.

Group	χ^2^	*p*-Value
T2DM	0.22	0.64
Prediabetes	0.24	0.62
Control	0.011	0.91

**χ^2^**: chi-square value.

**Table 4:** Shows Pairwise comparisons (alleles and Genotypes) between groups.

T2DM vs. Control				Prediabetes vs. Control			T2DM vs. Prediabetes		
	T2DM *n* (%)	Control *n* (%)	*p*-Value	Prediabetes *n* (%)	Control *n* (%)	*p*-Value	T2DM *n* (%)	Prediabetes *n* (%)	*p*-Value
Genotype									
TT	21 (10.5%)	2 (1.7%)	<0.001 *	8 (8%)	2 (1.7%)	<0.001 *	21 (10.5%)	8 (8%)	0.45
CT	92 (46%)	28 (23.3%)		44 (54%)	28 (23.3%)		92 (46%)	44 (54%)	
CC	87 (43.5%)	90 (75%)		48 (38%)	90 (75%)		87 (43.5%)	48 (38%)	
Allele									
T	134 (33.5%)	32 (13.3%)	<0.001 *	60 (30%)	32 (13.3%)	<0.001 *	134 (33.5%)	60 (30%)	0.4
C	266 (66.5%)	208 (86.7%)		140 (70%)	208 (86.7%)		266 (66.5%)	140 (70%)	

*: significant at *p* < 0.001. *n*: number.

**Table 5:** The association of TCF7L2 (rs7903146) with risk factors, including Clinical, Demographic, and Biochemical profiles, across all study groups.

Item	T2DM Cases (200)					Prediabetes Cases (100)				
	Status (*n*)	TT (*n* = 21)	CT (*n* = 92)	CC (*n* = 87)	*p*-Value	Status (*n*)	TT (*n* = 8)	CT (*n* = 44)	CC (*n* = 48)	*p*-Value
Clinical and demographic										
Nephropathy	Yes (17)	2	8	7	0.97					
	No (183)	19	84	80						
Retinopathy	Yes (11)	1	4	6	0.75					
	No (189)	20	88	81						
Cardiovascular diseases	Yes (15)	5	4	6	0.009 *					
	No (185)	16	88	81						
Neuropathy	Yes (8)	1	3	4	0.88					
	No (192)	20	89	83						
Hypertension	Yes (65)	7	30	28	0.99	Yes (22)	2	8	12	0.71
	No (135)	14	62	59		No (78)	6	36	36	
Smoking	Yes (71)	9	32	30	0.75	Yes (39)	3	17	19	0.99
	No (129)	12	60	57		No (61)	5	27	29	
BMI categories										
Normal	51	3	20	28	0.09	3	0	1	2	0.51
Overweight	139	15	68	56		88	6	40	42	
Obese	10	3	4	3		9	2	3	4	
Male	129	17	57	55	0.25	69	6	30	33	0.92
Female	71	4	35	32		31	2	14	15	
Biochemicals										
		Mean ± SD					Mean ± SD			
FPG (mmol/L)		7.4 ± 1.1	7.1 ± 0.5	7 ± 0.5	0.02 *		6.9 ± 1.1	6.6 ± 0.9	6.5 ± 0.2	0.043 *
HbA1c (%)		7.6 ± 1.1	7.5 ± 1.1	7.1 ± 0.9	0.016 *		6.1 ±0.2	6 ± 0.5	5.9 ± 0.2	0.045 *
TC (mmol/L)		4.22 ± 0.2	4.16 ± 0.6	4.1 ± 0.4	0.52		4 ± 0.2	3.98 ± 0.6	3.92 ± 0.2	0.67
TG (mmol/L)		1.8 ± 0.2	1.76 ± 0.6	1.67 ± 0.1	0.24		1.51 ± 0.1	1.43 ± 0.6	1.44 ± 0.1	0.72
LDL (mmol/L)		2.6 ± 0.2	2.58 ± 0.4	2.51 ± 0.1	0.19		2.39 ± 0.2	2.35 ± 0.2	2.31 ± 0.1	0.3
HDL (mmol/L)		1.16 ± 0. 1	1.17 ± 0. 1	1.19 ± 0. 1	0.28		1.18 ± 0.1	1.21 ± 0.1	1.23 ± 0.1	0.09
SOD (U/mL)		85.5 ± 12.9	86.3 ± 15.2	90.9 ± 10.1	0.03 *		110.9 ± 20.1	112.9 ± 18.6	115.9 ± 12.9	0.56
GPx (U/L)		41.9 ± 5.2	42.1 ± 6.9	45.1 ± 10.7	0.05		48.1 ± 12.2	49.5 ± 10.1	52.2 ± 13.7	0.46
TAC (mmol/L)		0.82 ± 0.2	0.99 ± 0.3	1.01 ± 0.3	0.027 *		1.07 ± 0.1	1.2 ± 0.1	1.29 ± 0.21	0.64
MDA (nmol/mL)		5.1 ± 0.6	4.5 ± 1.2	4.3 ± 1.1	0.013 *		4.7 ± 0.2	4.3 ± 0.6	4.1 ± 0.9	0.08

*: significant at *p* < 0.05. *n*: number. SD: standard deviation.

**Table 6:** *TCF7L2* (rs7903146) polymorphism and T2DM risk across several genetic models, including *p*-values, odds ratios (ORs), and 95% confidence intervals (CIs).

Model	Comparison	OR	95% CI	*p*-Value
Allelic	T vs. C	3.27	2.14–5.01	<0.001 *
Dominant	TT + CT vs. CC	3.9	2.37–6.42	<0.001 *
Recessive	TT vs. CT + CC	6.92	1.59–30.07	0.002 *

*: significant at *p* < 0.01. OR: adjusted odds ratio. CI: Confidence intervals.

**Table 7:** Multivariable logistic regression analysis evaluated variables linked to T2DM adjustment for age, BMI, sex, smoking status, hypertension, and hyperlipidemia.

Variables	OR	95% CI	*p*-Value
TT + CT genotype	3.75	2.60–5.51	<0.001 *
Age	1.12	0.15–2.21	0.89
BMI	2.16	0.98–3.99	0.056
Male	1.96	0.81–3.75	0.13
Smoking	2.01	0.87–3.80	0.10
Hypertension	1.82	0.69–3.65	0.22
Hyperlipidemia	2.91	1.81–4.89	<0.001 *

*: significant at *p* < 0.01. OR: adjusted odds ratio. CI: Confidence intervals.

**Table 8:** Multivariable linear regression investigation of the relationship between oxidative stress biomarkers and the TCF7L2 rs7903146 dominant genetic model (TT + CT vs. CC).

Biomarker	Adjusted β Coefficient	95% CI	*p*-Value
SOD	−7.92	−12.80 to −3.60	<0.001 *
GPx	−2.11	−4.39 to 0.31	0.08
TAC	−0.12	−0.17 to −0.03	0.006 *
MDA	0.49	0.23 to 0.89	0.002 *

*: significant at *p* < 0.01. β: unstandardized regression coefficient; CI: Confidence interval. Regression models were adjusted for age, sex, BMI, HbA1c, smoking status, hypertension, and diabetes duration. The dominant genetic model (TT + CT vs. CC) was used. *p* < 0.01 was considered statistically significant.

## Data Availability

Data used in this study are available upon request from the corresponding author; they are not public due to participant privacy.

## References

[1] Sen P., Alam S.S., Jahan I., Saha P., Akter F., Shill L.C. (2025). Association of the TCF7L2 rs7903146 variant with type 2 diabetes mellitus and related biomarkers: A systematic review. BMJ Nutr. Prev. Health.

[2] Hosseinpour-Niazi S., Mirmiran P., Hosseini S., Hadaegh F., Ainy E., Daneshpour M.S., Azizi F. (2022). Effect of TCF7L2 on the relationship between lifestyle factors and glycemic parameters: A systematic review. Nutr. J..

[3] Ning B., Wang J., Li B., Lyu C. (2022). Association of the transcription factor 7-Like 2 (TCF7L2) rs7903146 polymorphism with the risk of diabetic nephropathy: A meta-analysis. Horm. Metab. Res..

[4] Rani V., Deep G., Singh R.K., Palle K., Yadav U.C. (2016). Oxidative stress and metabolic disorders: Pathogenesis and therapeutic strategies. Life Sci..

[5] Fang C., Wu S., Zhang J., Tian Q., Zhang Z., Wu L. (2024). Impaired glucolipid metabolism in gestational diabetes mellitus with T variation of TCF7L2 rs7903146: A case–control study. Int. J. Diabetes Dev. Ctries..

[6] American Diabetes Association Professional Practice Committee (2025). 2. Diagnosis and Classification of Diabetes: Standards of Care in Diabetes-2025. Diabetes Care.

[7] Ahmed A.M., Al-Nakhle H.H., Aman A.M., Yousuf A.M., Muddathir A.R.M., Almutawif Y.A., Eweda S.M., Alsubhi A.S., Aljohani H.M., Alhamawi R.M. (2026). Association of NLRP3 Inflammasome rs10754558 Polymorphism with Type 2 Diabetes Mellitus and Its Complications: Clinical and Bioinformatics Study. Diabetes Metab. Syndr. Obes..

[8] Cao H., Song L., Wang X., Guan H. (2025). Non-linear relationship between urinary creatinine and diabetic kidney disease: Implications for clinical practice. BMC Nephrol..

[9] Flaxel C.J., Adelman R.A., Bailey S.T., Fawzi A., Lim J.I., Vemulakonda G.A., Ying G.-S. (2020). Diabetic retinopathy preferred practice pattern^®^. Ophthalmology.

[10] Elafros M.A., Callaghan B.C. (2023). Diabetic neuropathies. Contin. Lifelong Learn. Neurol..

[11] Nuha A., Rozalina M., Grazia A., Kirthikaa B., Elizabeth B., Kathaleen E., Dennis B., Sandeep D., Laya E., Rajesh G. (2025). 10. Cardiovascular disease and risk management: Standards of care in diabetes—2025. Diabetes Care.

[12] Lu Y.-C., Yu T.-H., Hsuan C.-F., Hsu C.-C., Hung W.-C., Wang C.-P., Tang W.-H., Cheng M.-C., Chung F.-M., Lee Y.-J. (2025). Association of TCF7L2 rs7903146 (C/T) Polymorphism with Type 2 Diabetes Mellitus in a Chinese Population: Clinical Characteristics and Ethnic Context. Diagnostics.

[13] Hu S., Ji W., Zhang Y., Zhu W., Sun H., Sun Y. (2025). Risk factors for progression to type 2 diabetes in prediabetes: A systematic review and meta-analysis. BMC Public Health.

[14] Choo Y.N., Ravi R.N., Subramaniyan V. (2026). Insulin resistance induced by obesity: Mechanisms, metabolic implications and therapeutic approaches. Mol. Biol. Rep..

[15] Mlonga J., Damian D. (2025). Lipid profile abnormalities in type 2 diabetes mellitus patients at Mbeya Zonal Referral Hospital, Tanzania: A retrospective study. J. Diabetes Res..

[16] Bennici G., Almahasheer H., Alghrably M., Valensin D., Kola A., Kokotidou C., Lachowicz J., Jaremko M. (2024). Mitigating diabetes associated with reactive oxygen species (ROS) and protein aggregation through pharmacological interventions. RSC Adv..

[17] Caturano A., D’Angelo M., Mormone A., Russo V., Mollica M.P., Salvatore T., Galiero R., Rinaldi L., Vetrano E., Marfella R. (2023). Oxidative Stress in Type 2 Diabetes: Impacts from Pathogenesis to Lifestyle Modifications. Curr. Issues Mol. Biol..

[18] Weinberg Sibony R., Segev O., Dor S., Raz I. (2024). Overview of oxidative stress and inflammation in diabetes. J. Diabetes.

[19] Mahajan A., Spracklen C.N., Zhang W., Ng M.C., Petty L.E., Kitajima H., Yu G.Z., Rüeger S., Speidel L., Kim Y.J. (2022). Multi-ancestry genetic study of type 2 diabetes highlights the power of diverse populations for discovery and translation. Nat. Genet..

[20] Szczerbinski L., Florez J.C. (2023). Precision medicine of obesity as an integral part of type 2 diabetes management–past, present, and future. Lancet Diabetes Endocrinol..

[21] Choi Y., Kwon H.-K., Park S. (2023). Polygenic variants linked to oxidative stress and the antioxidant system are associated with type 2 diabetes risk and interact with lifestyle factors. Antioxidants.

[22] Sousa A.G.P., Marquezine G.F., Lemos P.A., Martinez E., Lopes N., Hueb W.A., Krieger J.E., Pereira A.C. (2009). TCF7L2 polymorphism rs7903146 is associated with coronary artery disease severity and mortality. PLoS ONE.

[23] Hansen A.L., Andersen M.K., Engelhard L.M., Brøns C., Hansen T., Nielsen J.S., Vestergaard P., Højlund K., Jessen N., Olsen M.H. (2025). Impact of TCF7L2 rs7903146 on clinical presentation and risk of complications in patients with type 2 diabetes. Diabetes Obes. Metab..

[24] Caturano A., Rocco M., Tagliaferri G., Piacevole A., Nilo D., Di Lorenzo G., Iadicicco I., Donnarumma M., Galiero R., Acierno C. (2025). Oxidative stress and cardiovascular complications in type 2 diabetes: From pathophysiology to lifestyle modifications. Antioxidants.

[25] Ghosheh N., Naffa R.G., Mashal S., Al-Khalayfa S., Battah A.H., Azab B. (2025). The association of TCF7L2 gene polymorphisms, rs12255372 and rs7903146, with type 2 diabetes mellitus in the Jordanian population. Mol. Biol. Rep..

[26] Grant S.F., Thorleifsson G., Reynisdottir I., Benediktsson R., Manolescu A., Sainz J., Helgason A., Stefansson H., Emilsson V., Helgadottir A. (2006). Variant of transcription factor 7-like 2 (TCF7L2) gene confers risk of type 2 diabetes. Nat. Genet..

[27] Florez J.C. (2007). The new type 2 diabetes gene TCF7L2. Curr. Opin. Clin. Nutr. Metab. Care.

[28] Lyssenko V., Lupi R., Marchetti P., Del Guerra S., Orho-Melander M., Almgren P., Sjögren M., Ling C., Eriksson K.-F., Mancarella R. (2007). Mechanisms by which common variants in the TCF7L2 gene increase risk of type 2 diabetes. J. Clin. Investig..

[29] Gaulton K.J. (2017). Mechanisms of type 2 diabetes risk loci. Curr. Diabetes Rep..

[30] Evans J.L., Goldfine I.D., Maddux B.A., Grodsky G.M. (2002). Oxidative stress and stress-activated signaling pathways: A unifying hypothesis of type 2 diabetes. Endocr. Rev..

[31] Gaulton K.J., Nammo T., Pasquali L., Simon J.M., Giresi P.G., Fogarty M.P., Panhuis T.M., Mieczkowski P., Secchi A., Bosco D. (2010). A map of open chromatin in human pancreatic islets. Nat. Genet..

[32] Pasquali L., Gaulton K.J., Rodríguez-Seguí S.A., Mularoni L., Miguel-Escalada I., Akerman I., Tena J.J., Morán I., Gómez-Marín C., Van De Bunt M. (2014). Pancreatic islet enhancer clusters enriched in type 2 diabetes risk-associated variants. Nat. Genet..

[33] Parker S.C., Stitzel M.L., Taylor D.L., Orozco J.M., Erdos M.R., Akiyama J.A., Van Bueren K.L., Chines P.S., Narisu N., Program N.C.S. (2013). Chromatin stretch enhancer states drive cell-specific gene regulation and harbor human disease risk variants. Proc. Natl. Acad. Sci. USA.

[34] Welters H.J., Kulkarni R.N. (2008). Wnt signaling: Relevance to β-cell biology and diabetes. Trends Endocrinol. Metab..

[35] Nusse R., Clevers H. (2017). Wnt/β-catenin signaling, disease, and emerging therapeutic modalities. Cell.

[36] Pinti M.V., Fink G.K., Hathaway Q.A., Durr A.J., Kunovac A., Hollander J.M. (2019). Mitochondrial dysfunction in type 2 diabetes mellitus: An organ-based analysis. Am. J. Physiol.-Endocrinol. Metab..

